# Demand, End-Uses, and Conservation of Alpine Medicinal Plant* Neopicrorhiza scrophulariiflora* (Pennell) D. Y. Hong in Central Himalaya

**DOI:** 10.1155/2018/6024263

**Published:** 2018-06-19

**Authors:** Gandhiv Kafle, Indira Bhattarai (Sharma), Mohan Siwakoti, Arjun Kumar Shrestha

**Affiliations:** ^1^Faculty of Agriculture, Agriculture and Forestry University, Chitwan, Nepal; ^2^Central Department of Botany, Tribhuvan University, Kathmandu, Nepal

## Abstract

*Neopicrorhiza scrophulariiflora* (Pennell) D. Y. Hong of Scrophulariaceae family (hereinafter referred to as* Neopicrorhiza*) has medicinally important rhizomes with high levels of trade. What factors drive demand for* Neopicrorhiza* in Central Himalaya is unknown. In this context, a nationwide comprehensive survey was conducted from September 2016 to March 2017 to assess demand, end-uses, and conservation of dry* Neopicrorhiza* rhizomes in Nepal. A total of 2313 herbal products were surveyed for* Neopicrorhiza* as an ingredient in 38 retailer shops. Processing industries of* Neopicrorhiza* in Nepal were interviewed using structured questionnaire. There were 23 herbal industries manufacturing 45 types of ayurvedic medicines as end-products containing* Neopicrorhiza*. The volume and value of annual demand for dry rhizomes of* Neopicrorhiza* in Nepal were found as 6076 kg and NRs 8573236 (USD 83235.30), respectively, in 2015/016 with average 264.17 kg/industry and NRs 1410.87 (USD 13.69) per kg. The major uses of ayurvedic medicines containing* Neopicrorhiza* were to treat a number of disease categories: cardiovascular system/liver (17), cardiovascular system/blood (6), nervous system (6), dermatological system (4), musculoskeletal system (3), digestive system (2), respiratory system (2), genitourinal system (4), and others (1). Despite changing legal regulation, trade and consumption of* Neopicrorhiza *exist in Nepal. It can be concluded that domestic consumption is not the major cause of resource depletion of* Neopicrorhiza* in Nepal.

## 1. Introduction

*Neopicrorhiza scrophulariiflora* (Pennell) D. Y. Hong (Scrophulariaceae) (hereinafter referred to as* Neopicrorhiza*) is a perennial alpine herb found in the subalpine as well as alpine zone of the eastern Himalayas comprising Sikkim, Nepal, Bhutan, and China [1–3]. It occurs in the wild in diverse habitat types: alpine grassland and gravelly areas, forests, shrublands, meadows, cliffs and screes, between 3600 and 4400 masl [4–6]. It prefers moist north-facing slopes with richer and partial shady soil [6]. Its Nepali name is Kutki, which is assessed as being vulnerable in Nepal [7].* Neopicrorhiza* is prioritized by Government of Nepal for research and economic development among 30 medicinal and aromatic plants [8].

The long, creeping, and bitter rhizomes of* Neopicrorhiza* are used medicinally. Its rhizomes have been officially listed in the China's Pharmacopoeia for the treatment of fever, jaundice, hemorrhoids, and dysentery by Traditional Chinese and Tibetan Medicine [9]. In Nepal, a number of ethnobotanical uses of its unprocessed rhizomes have well been documented to treat common cold, fever, sinusitis, headache, diarrhea, paralysis, hysteria, anemia, high blood pressure, sore throat, gastritis, intestinal pains, snake and scorpion sting, abdominal pain, indigestion, liver troubles, bile disorders, continuous pain in the chest and heart, increased heartbeat, difficulty in breathing, jaundice, cuts, wounds, and conjunctivitis [10–18]. Chemical constituents of rhizomes of* Neopicrorhiza* are well studied which comprise iridoid glycosides [19–21], triterpenoids [22], phenolic glycosides [23], phenylethanoid glycosides [24], cucurbitacin glycoside [25, 26], and phenylpropanoid glycosides [27] having a wide range of biological activities.

The dry and unprocessed rhizomes of* Neopicrorhiza* are under commercial trade. Though Nepal supplied 66 ± 12% of rhizomes of* Neopicrorhiza *in the global supply chain [30], indicating higher levels of harvesting, the domestic demand for this species in Nepal is unknown. Shrestha and Jha [6] reported that populations of* Neopicrorhiza* are fragmented and its population sizes appear to be declining despite no commercial collection at study period in trans-Himalayan dry valley of central Nepal, Manang district. Shrestha and Shrestha [31] reported unsustainable harvesting method of* Neopicrorhiza* in Rasuwa district of northern Nepal, where the plant was entirely uprooted with no part left for future regeneration. Ghimire et al. [5] reported that the size class of rhizomes of* Neopicrorhiza* harvested for trade was significantly smaller than those collected for health care, leading to premature harvesting. Sustainable harvesting of underground parts (rhizomes, roots, bulbs, or other storage organs) of long-lived species presents a particularly great challenge [32]. The high levels of trade and unsustainable harvesting can lead to pressure on the growing stock of* Neopicrorhiza* in Nepal, though currently national population status of this species is lacking. A rising demand for Himalayan plant based medicinal and cosmetic products was mentioned by Holley and Cherla [33] and Olsen [34] which could foster demand for raw materials, which hence could lead to rise in harvest level of medicinal and aromatic plant species. So it has become important to understand what is driving the demand for* Neopicrorhiza* in Nepal. Understanding end-uses of plant species is essential for prediction of future demand for the species and planning for its harvesting sustainability. However, nothing is known about the industrial consumption and end-products of* Neopicrorhiza*. The aim of this paper is to contribute to the understanding of the end-uses of commercially important medicinal plants by exploring the industrial demand for rhizomes of* Neopicrorhiza* and its end-products, uses, and conservation in Nepal.

## 2. Materials and Methods

Step 1. Telephone survey: First of all, a list of herbal manufacturing industries in Nepal was compiled during September–November 2016 using a number of tools—web survey, online database analysis of Department of Plant Resources of Government of Nepal, database analysis of Department of Industry of Government of Nepal, analysis of directory of national census of manufacturing establishments (2069/2011), directories of herbal associations in Nepal, and stakeholder consultation. The output was a list of 246 herbal manufacturing industries.

Telephone interview adapted from Stanton and Futrell [35], Cooper and Schindler [36], and Boyd et al. [37] was conducted with representative of herbal manufacturing industries in November 2016 to find out the industries that process* Neopicrorhiza*. The main tool for the data collection with “telephone survey” was a structured questionnaire, finalized by expert panel and pretesting, and was composed of two major sections.  Section 1 included introduction to the topic and researcher.  Section 2 included categories of end-products the industry is currently producing, whether the industry processes* Neopicrorhiza* or not, name, location, and contact details of the industry.

Step 2: Market survey: The herbal products for* Neopicrorhiza* as an ingredient were randomly surveyed at 2313 herbal products at retailer shops in two big cities of Nepal, during November–December 2016 (see Table 1). We surveyed 1286 herbal products in Kathmandu city in 30 shops and 1027 herbal products at 8 retailer shops of Bharatpur city of Chitwan district of Nepal.

The main tool for the data collection in “market survey” was a structured questionnaire, finalized by expert panel and pretesting, and was composed of introduction, name of end-product containing* Neopicrorhiza*, form of product, manufacturers' details, who mainly buys, basis of sale (prescription or without prescription), use of product, and retail price.

Step 3. Industrial survey: A total of 23 industries processing* Neopicrorhiza* were identified in Nepal and visited during January–March 2017, and face-to-face interviews were conducted with representatives of these industries using structured questionnaire, finalized by expert panel and pretesting.

The structured questionnaire was composed of three major sections.  Section 1 included characteristics of respondents such as name, age, education, and number of years of experiences in processing of medicinal plants.  Section 2 included characteristics of the industry such as name, year of establishment, and number of regular staff.  Section 3 included name of end-product or end-product containing* Neopicrorhiza*, amount of its raw rhizomes needed, price paid for its raw rhizomes, and marketing channel of end-products. The information on end-products collected from market survey was also cross-checked and validated with this survey.

Respondents were informed about the purpose of the research and their prior consent was obtained for interview. The research did not require approval of ethics from government of Nepal since it was a pure academic research mainly based on noninvasive and nondestructive methods (i.e., interviews).

The standard terminology for categories of diseases was adopted from Kunwar et al. [29]. Data analysis was performed using MS Excel and IBM Statistics SPSS 20. Descriptive statistics including frequency, percentage, mean, sum, and average were used. The information was presented in tables and explained.

## 3. Results and Discussion

### 3.1. Characteristics of Processing Industries of* Neopicrorhiza* in Nepal

**Figure 1:**
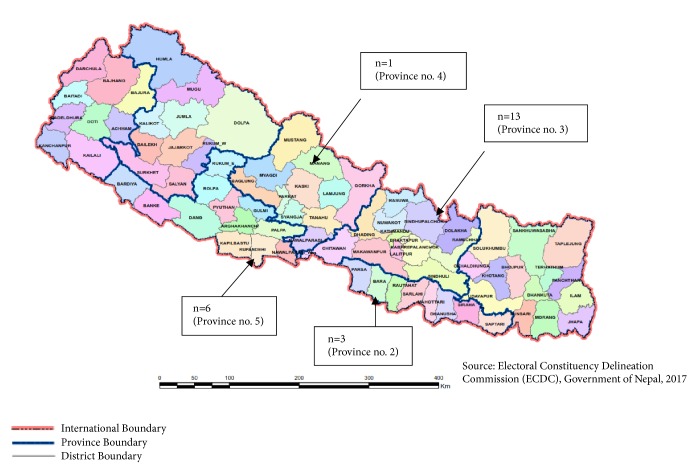
Distribution of* Neopicrorhiza*-processing industries in seven provinces of Nepal. Note:* The Provinces of Nepal were formed on 20 September 2015 according to Schedule 4 of the Constitution of Nepal. According to Article 295 (2), the permanent names of the provinces will be determined by a 2/3 vote of the respective province's legislature. They were officially indicated as 1,2,3,4,5, and 7 provinces. Province number 6 became the first province to get a name, as a provincial assembly meeting renamed it “Karnali” on 24 February 2018*.

Out of 246 herbal manufacturing industries, a total of 23 industries were found processing dry rhizomes of* Neopicrorhiza* in Nepal to manufacture its end-products. The number of* Neopicrorhiza*-processing industries in province three was the highest number of industries (n=13, 56.52%), while province four had the lowest number of those industries (n=1, 4.35%). The number of* Neopicrorhiza*-processing industries in provinces two and five was three (13.04%) and six (26.09%), respectively. There was not any* Neopicrorhiza*-processing industries in province number 1, Karnali province, and province number 7 (Figure 1). Out of the 23* Neopicrorhiza*-processing industries recorded, 8 (34.78%) were located in Kathmandu district, 6 (26.09%) in Rupandehi district, and two (8.69%) in each of Parsa, Lalitpur (8.69%), and Makawanpur districts, and one (4.35%) was located in each of Chitwan, Gorkha, and Dhading districts. Herbal processing and manufacturing are still in their infancy in Nepal, though herbal industries have a great scope of providing employment and developing new natural products that can fetch better prices in international markets [38]. Winrock International et al. [39] identified only 15 major companies involved in the processing of 205 non-timber forest products for making ayurvedic preparations and essential oils. Edwards [40] had projected that Nepal will find it difficult to compete with India's highly complex, professionally managed herbal processing operations, which are on a considerably larger scale than may ever be developed in Nepal. We argue that there is a definite need for nation's priority towards the expansion of ayurvedic medicine and herbal manufacturing industries and to create conducive environment for competitive functioning in Nepal.

Characteristics of the industries that involved processing rhizomes of* Neopicrorhiza* are summarized in Table 2. All industries were established between the periods of 1963 and 2013. Except Singha Darbar Vaidhya Khana, all were privately owned (n=22, 95.65%). Majority of the industries (n=20, 86.95%) were established after 1992. The industries had different number of regular staff ranging from 2 to 50. Most of the industries (n=11; 47.83%) had 2–11 regular staff. The scales of* Neopicrorhiza*-processing industries were of small (30.43%), medium (34.78%), and large (34.78%) as perceived and reported by the respondents themselves.

Our research shows that majority of the* Neopicrorhiza*-processing industries of Nepal emerged after 1992 AD, privately owned except one government owned Singha Darbar Vaidhya Khana, started during Rana regime for royal palace. The Industrial Enterprises Act of 1974 and its frequent amendments shifted the government's emphasis on growth from the public to the private sector in Nepal [41]. Nepal entered into multiparty democratic system in 1990 after a nationwide successful revolution, which increased private sector development in the country, including industries. The Industrial Policy of Nepal came into effect only in 2010 that identified priority industries for Nepal, including forest based industries, and ayurvedic and homeopathic medicine manufacturing, as priority industries [42].

### 3.2. Domestic Industrial Consumption (Volume and Value) of* Neopicrorhiza *in Nepal

The domestic industrial demand for* Neopicrorhiza* in Nepal was unknown for a long time. Industrial survey shows that the annual demand for dry rhizomes of* Neopicrorhiza* in* Neopicrorhiza*-processing industries (23) in Nepal was 6076 kg (6.076 tons). It is novel finding. The demand for its rhizomes ranged from 5 to 1000 kg/yr in different industries, with average of 264.17 kg/yr per industry. We compared our findings with that of India where the annual domestic consumption of* Picrorhiza kurroa* was 415 MT [43]. Rhizomes of* Picrorhiza kurroa* (found in western Himalaya) and* Neopicrorhiza scrophulariiflora* (found in eastern and central Himalaya) are morphologically similar and enter into trade without differentiation. The annual legal trade of* Neopicrorhiza* in Nepal in fiscal year 2015/016 was 61 tons [44] which is around ten times higher than the domestic industrial demand for this species (6 tons) in 2015/016 as found in this study. Olsen [34] indicated with no doubt that the annual harvested amounts far exceed the domestic demand for medicinal and aromatic plants in Nepal, and we also found similar results for* Neopicrorhiza*. Olsen [30] estimated 100–400 tons of annual* Neopicrorhiza* export from Nepal. Though the export figures vary, these data indicate higher levels of export of dry* Neopicrorhiza* rhizomes from Nepal than domestic consumption. Province-wise annual demand for* Neopicrorhiza* in industries of Nepal in 2015/016 is presented in Table 3, with highest demand in province three and lowest in province four. None of the herbal industries located in provinces 1, 6 (hereinafter referred as Karnali), and 7 were found using* Neopicrorhiza* as raw material.

The industries paid NRs 1150 to 1600 (USD 11.16–15.53) to purchase one kilogram of dry rhizomes of* Neopicrorhiza* in 2015/016, with average price of NRs 1410.87 (USD 13.69) per kg. The latest royalty rate for one kilogram of* Neopicrorhiza* rhizomes is NRs 15 (USD 0.145) [45], indicating too much margin in trade to be paid by industries for* Neopicrorhiza* rhizomes. Olsen [30] reported that local traders paid NRs 87.9 (USD 0.853) in average to purchase one kilogram of dry rhizomes of* Neopicrorhiza* in 1997/98. Total annual value of dry rhizomes of* Neopicrorhiza* purchased for domestic industrial consumption in Nepal in 2015/016 was found NRs 8573236 (USD 83235.30) for 6.076 tons, which is much lesser than Olsen's estimate of 1.5 million USD annual export value of dry rhizomes of* Neopicrorhiza* from Nepal in 1997/98.

### 3.3. Availability of* Neopicrorhiza* for Industries and Its Substitutes

All the respondents reported that their industries had not faced any difficulty in obtaining quality rhizomes of* Neopicrorhiza* to fulfill their annual demand. In most of the cases (18, 78.26%), the traders themselves made contact with the industries and provided sample of dry rhizomes of* Neopicrorhiza*. Then the specialists in industries inspected the quality of the rhizomes and made decision whether to purchase or not. They did not depend on single trader for purchase of the dry rhizomes of* Neopicrorhiza*. Only 6 (21.74%) industries contacted traders from their list to purchase the dry rhizomes of* Neopicrorhiza*. In both cases, price of the rhizomes was also the major competitive factor while deciding the purchase from particular trader. All the* Neopicrorhiza*-processing industries did not use any substitute for* Neopicrorhiza*, indicating that their demand for its rhizomes were easily fulfilled, they did not require large quantities of* Neopicrorhiza* annually, and* Neopicrorhiza* was only used as an ingredient in end-products. Plants gathered in Nepal are used either for domestic consumption or for sale and manufacturing the end-products in Nepal, India, and third countries [46], as also found in this study. The lower levels of demand for* Neopicrorhiza* and government's lifting of ban for collection and domestic trade might be the main reasons for ease in obtaining quality rhizomes of* Neopicrorhiza* by the industries.

*Picrorhiza kurroa*, of which India is a main consumer with annual domestic consumption of 415 MT [43], is open for trade in India. However, Olsen [30] argued that the majority of trade occurs in* Neopicrorhiza* from Nepal rather than* Picrorhiza kurroa *in India. Demand for* Picrorhiza kurroa* is continuously increasing in India; e.g., an annual growth rate of 12.9% was recorded for its demand for 220 tons in 2001-2002 and 317 tons during 2004-2005 [47]. China is apparently a major consumer of* Neopicrorhiza* [34]. This scenario indicates that there will definitely be pressure on supply status of* Neopicrorhiza* in future to industries due to this increasing regional demand. Multiple uses exert higher demand, leading to increased harvest, and such actions raise threats for medicinal plant species [18]. Regional level strategies will be necessary to keep balance between trade and conservation of* Neopicrorhiza*. Till date, population status of* Neopicrorhiza* in Nepal is unknown, without which any assumption of sustainable supply and trade of this species on long run might be misleading.

### 3.4. End-Products of* Neopicrorhiza*

We did not know before what end-products were manufactured from* Neopicrorhiza*. Market and industrial surveys revealed that* Neopicrorhiza* was used for manufacturing forty-five (45) herbal products in Nepal. A complete list of those end-products with description is presented in Table 4. All those end-products were ayurvedic medicines.* Neopicrorhiza* was used only as an ingredient in these products. The end use of* Neopicrorhiza* for production of only ayurvedic medicines can be explained by the presence of medicinally useful iridoid glycosides such as picrosides I and II, and kutkoside in its rhizomes [48–52]. Out of 45 ayurvedic medicines containing* Neopicrorhiza*, 15 (33.33%) were classical (Classical Ayurvedic Medicine (CAM) is prepared by considering standard formulations from traditional Ayurvedic text books like Charaka Samhita, Sushruta Samhita, etc. Those formulations remain the same for specific medicines irrespective of the manufacturers.) and 30 (66.67%) were proprietary (Proprietary Ayurvedic Medicine (PAM) is prepared by industries using own formulations.) medicines. The forms of these medicines were different: 6 (13.33%) were in tablet form, 18 (40%) were in syrup form, 15 (33.33%) were in powder form, 5 (11.11%) were in gel form, and 1 (2.22%) was in capsule form.

The annual consumption of dry rhizomes of* Neopicrorhiza* for production of Hepadex (a liver tonic) was recorded as the highest (1000 kg). The annual consumption of dry rhizomes of* Neopicrorhiza* for production of particular end-product containing* Neopicrorhiza* ranged from 2 to 1000 kg. The average annual consumption of dry rhizomes of* Neopicrorhiza* for production of each product is 135.02 kg. The top three products, for production of which more than 500 kg dry rhizomes of* Neopicrorhiza* were purchased in a year, were Hepadex (a liver tonic), Rohitkyadi Churna (a liver medicine), and Hepagard DS (a liver tonic).

### 3.5. Production Rate of End-Products of* Neopicrorhiza* Adopted by Industries

The number of industries producing particular product and rank of production of each product is presented in Table 4. Rohitkyadi Churna, Neembadi Churna, and Arogyavardini Vati are the top three end-products containing* Neopicrorhiza* in terms of production by higher number of industries. Among these products, Rohitkyadi Churna enjoyed the highest production rate of 14.5%. Edwards [40] argued that the technology behind much processing of non-timber forest products was relatively straightforward and a wide range of ayurvedic preparations was already produced in Nepal, albeit on a small scale. Our findings on the data of annual demand for dry* Neopicrorhiza* rhizomes in domestic industries (6.076 tons) and their expense on these rhizomes (USD 83235.30) is consistent with Edwards's general view of small scale production of ayurvedic medicines within Nepal.

Nepali and Indian manufacturers of Ayurvedic medicines available in the Nepalese markets are nearly equal in number, but the Indian products are dominating the Nepalese market [53]. It is interesting to note that, out of 45* Neopicrorhiza*-containing ayurvedic medicines, only three of those medicines were produced by more than one industry (Table 4). It shows that industries of Nepal have not yet diversified the* Neopicrorhiza*-containing ayurvedic medicines in their production system. More processing of plants and the production of end-products in Nepal would greatly increase the value of the plants to the national economy [54]. Around only ten percent herbal industries out of 246 surveyed herbal industries in Nepal were found processing* Neopicrorhiza* rhizomes to produce the end-products. Hence, when issues emerge on resource depletion of* Neopicrorhiza* in Nepal, we here argue that it is due to export of* Neopicrorhiza* from Nepal than from domestic consumption, and regulatory measures should address the trade of* Neopicrorhiza* in terms of foreign export.

### 3.6. Uses of End-Products of* Neopicrorhiza*

We found that* Neopicrorhiza* is used to produce only Ayurvedic medicines. The major uses of the ayurvedic medicines containing* Neopicrorhiza* were recorded to treat a number of illness categories: cardiovascular, nervous, dermatological, musculoskeletal, genitourinal, respiratory, digestive, and others. The highest number of products (n=17, 37.78) was reported to treat disease associated with liver. Previous studies had explored the use of the unprocessed rhizomes of* Neopicrorhiza* to treat various ailments such as liver disorders, fever, asthma, and jaundice and have pharmaceutical value for hepatoprotective, immunomodulator, and antiasthmatic activities [22, 55, 56] in Indian, Bhutanese, Tibetan, and Chinese traditional medicines. Mulliken and Crofton [57] argued that it is not inconceivable that in future the flow of* Picrorhiza/Neopicrorhiza* supplies could shift to the north (China), particularly if the efficacy of* Picrorhiza kurroa* in the treatment of liver disease is confirmed.

The information about major use (indication) of the ayurvedic medicine provided in the label of the medicines was cross-checked and validated during market survey and industrial survey and was found consistent with information of Table 5.

### 3.7. Legislation, Legal Trade, and Conservation of* Neopicrorhiza* in Nepal

Government of Nepal made a series of changes regarding the legal provisions for trade and conservation of the* Neopicrorhiza* (Table 6). In 2001, Government of Nepal banned for collection, utilization, sale, distribution, transport, and export of* Neopicrorhiza* [58]. In 2003, the government had revised the restriction based on Forest Act 1993 [59]. The government has set conditions for export of this species: (1) clear taxonomic identification of the* Neopicrorhiza* and its confirmation by the Department of Plant Resources are mandatory. (2) Department of Forest then permits the export after assessing the availability of the species.

Government of Nepal has changed the royalty rates of medicinal and aromatic plant products from time to time, including* Neopicrorhiza* [60]. In 2005, the royalty rate was NRs 10/kg [61]. The latest revision of the Forest Regulations was enforced in 2015 and it fixed a royalty rate of NRs 15/kg* Neopicrorhiza *[45]. In 2005, the government fixed NRs 500 as royalty for issuing CITES certificate for export of CITES listed species including* Neopicrorhiza *[61], which was increased to NRs 1000 in 2015 [45].

Despite the changes in legal provisions for trade and conservation of* Neopicrorhiza* from time to time, the trade of this species exists in Nepal. We analyzed the legal trade data of* Neopicrorhiza* of six fiscal years (2010–2016). The analysis of government data shows that 224.635 tons of* Neopicrorhiza* entered into legal trade during 2010–2016 (6 years period) in Nepal, with average of 37.44 tons/yr and the government collected a total of NRs 3360945 (USD 32630.53) from its royalty (Table 7). 33.652 tons of* Neopicrorhiza* was collected and traded from Humla district during the fiscal year 1999-2000 AD (2055-056 BS) [62]. The government's official records show that the total volume of dry rhizomes of* Neopicrorhiza* exported to India, after receiving CITES certificate, from Nepal during 2015/016 period was around 28 tons [44]. Excessive and unregulated commercial harvest of medicinal plants (legally and illegally) has caused direct threat to the high value species [63]. Apart from unsustainable harvesting, other factors such as debris deposition at pastureland and cliff, deforestation, habitat encroachment, overgrazing, wildfires, shifting cultivation, and climate change contribute to species loss [4, 17, 18, 29, 64].

Looking at the province-wise trade, the legally traded volumes of* Neopicrorhiza* in provinces 3,4, Karnali, and 7 were 1.43, 15.91, 184.34, and 22.94 tons, respectively, with the highest in Karnali province (184.34 tons) and the lowest in province three (1.43 tons). In 2015/016, the legally traded volume of* Neopicrorhiza* in Nepal was about 61 tons [44]; our research showed that the domestic industrial consumption of this species was only around 6 tons in 2015/016. The remaining amount (61-28-6 = 27 tons) might be exported to third countries and/or sold in local markets. In average, 12.2 tons of* Neopicrorhiza* was traded annually from Gorkha district in 1994/95 [34]. Olsen [30] estimated 100–400 tons of annual* Neopicrorhiza* export from Nepal in 1998/99. These estimates seemed much higher when compared to legally traded volume of* Neopicrorhiza* in 2015/016 (i.e., 61 tons). This variation could hint for possibility of existence of illegal trade of* Neopicrorhiza* through Nepal. The main challenge will be how to regulate the export of this species outside the country while meeting the domestic demand and maintaining sustainable growing stock of this species. However, currently the domestic consumption of* Neopicrorhiza* cannot be entitled to be the root cause of resource depletion of this species.

## 4. Conclusions

This research presents a first exploration of domestic demand for alpine medicinal plant* Neopicrorhiza*, and its end-uses and manufacturers in Nepal. The annual domestic demand for dry rhizomes of* Neopicrorhiza* in Nepal was found around six tons in case year 2015/016 and this value is lesser than that of total traded volume and export. Forty-five end-products were found containing* Neopicrorhiza* as an ingredient and all those products were ayurvedic medicines. The major uses of the ayurvedic medicines containing* Neopicrorhiza* were to treat diseases associated with cardiovascular, nervous, dermatological, musculoskeletal, genitourinal, respiratory, and digestive systems. It can be concluded that the domestic consumption is not the major cause of resource depletion of* Neopicrorhiza* in Nepal. Regional consideration is necessary in setting priorities for conservation of* Neopicrorhiza* and sustainable trade in and from Nepal. In addition, growing stock of this species should be quantified throughout its habitat to set such priorities. Similar studies on end uses of* Neopicrorhiza* are recommended in countries (e.g., India) where this species is exported from Nepal.

## Figures and Tables

**Table 1:** Details of herbal products surveyed for *Neopicrorhiza* as an ingredient in retailer shops (n=38).

**Herbal product categories** ^**∗**^	**Herbal products checked in Kathmandu**	**Herbal products checked in Bharatpur**	**Total herbal products checked**
Ayurvedic medicine	405	725	1130

Herbal cosmetics	162	75	237

Incense	67	26	93

Massage oil	67	59	126

Soap and shampoo	105	58	163

Unprocessed herb	84	0	84

Food additive	107	22	129

Vegetable oil	12	0	12

Tea	277	62	339

**Total**	**1286**	**1027**	**2313**

Source: Field survey (2015/16).

^*∗*^Herbal product categories mentioned in Table 1 were adopted from Smith-Hall et al. [28].

**Table 2:** Characteristics of the *Neopicrorhiza*-processing industries (n=23).

Variables	Categories	Frequency	Percentage
Year of establishment of industry	1963-1972	2	8.70%
	1983-1992	1	4.35%
	1993-2002	12	52.17%
	2003-2013	8	34.78%

Ownership of industry	Privately owned	22	95.65%
	State owned	1	4.35%

Number of full-time staff	2-11	11	47.83%
	12-21	1	4.35%
	22-31	3	13.04%
	32-41	1	4.35%
	42-51	7	30.43%

Self-reported size of industry	Large	8	34.78%
	Medium	8	34.78%
	Small	7	30.43%

**Table 3:** Province-wise industrial demand of *Neopicrorhiza* in 2015/16.

Province 2			Province 3			Province 4			Province 5		
Districts	Volume (tons)	Value (USD)	Districts	Volume (tons)	Value (USD)	Districts	Volume (tons)	Value (USD)	Districts	Volume (tons)	Value (USD)
Parsa	0.975	3375.72	Kathmandu	1.721	19422.33	Gorkha	0.340	5048.54	Rupandehi	2.090	24407.76
Rautahat	0.200	2184.46	Lalitpur	0.270	16669.90						
			Dhading	0.400	8388.35						
			Chitwan	0.050	792.23						
			Makawanpur	0.030	1310.68						
Total	1.175	5560.18		2.471	46583.49		0.340	5048.54		2.090	24407.76

Flat exchange rate: USD 1 = NRs 103; USD = United States Dollar, NRs = Nepalese Rupees.

**Table 4:** End-products of Neopicrorhiza with their processing industries, consumption, uses, and production rank in 2015/16.

**End product containing *Neopicrorhiza***	**Form**	**Number of processing industries**	**Consumption of dry *Neopicrorhiza* rhizomes (kg/yr)**	**Category of medicine**	**Disease category/use**	**Rank of production**
Rohitkyadi churna	Powder	8	619	CAM	CVC (liver)	1

Neembadi churna	Powder	3	259	CAM	DER	2

Arogyavardini vati	Tablet	2	250	CAM	NVS	3

Asthamarin	Tablet	1	20	PAM	RES	4

Ayurved shakti churna	Powder	1	2	PAM	NVS	5

Dardnasak oil	Syrup	1	150	PAM	MSK	6

Diabeno	Powder	1	8	PAM	CVC (blood)	7

Gastro	Powder	1	100	PAM	DIG	8

Glowderm	Syrup	1	200	PAM	CVC (blood)	9

Haridrakhanda	Powder	1	7	CAM	DER	10

Hepadex	Syrup	1	1000	PAM	CVC (liver)	11

Hepagard DS	Syrup	1	540	PAM	CVC (liver)	12

Hepatop	Syrup	1	50	PAM	CVC (liver)	13

Heptogen capsule	Capsule	1	40	PAM	CVC (liver)	14

Heptogen syrup	Syrup	1	90	PAM	CVC (liver)	15

Jameda churna	Powder	1	5	CAM	CVC (blood)	16

Jatyadi ghrita	Gel	1	2	PAM	DER	17

Jeevan shakti prash	Gel	1	30	PAM	NVS	18

Kasarin	Syrup	1	70	PAM	RES	19

Kumaryasava	Syrup	1	100	CAM	GUS	20

Kustadi danta manjan	Gel	1	100	PAM	OTH	21

Lachhadi oil	Syrup	1	20	CAM	GUS	22

Livergen	Syrup	1	150	PAM	CVC (liver)	23

Livherb	Syrup	1	200	PAM	CVC (liver)	24

Livorin	Syrup	1	100	PAM	CVC (liver)	25

Livosave	Syrup	1	300	PAM	CVC (liver)	26

Livotop	Syrup	1	400	PAM	CVC (liver)	27

Madhumeha churna	Powder	1	100	CAM	CVC (blood)	28

Maha sudarshan churna	Powder	1	2	CAM	CVC (blood)	29

Mahayogaraj guggul	Tablet	1	100	CAM	MSK	30

Mana	Gel	1	30	PAM	NVS	31

Megaferol	Gel	1	300	PAM	NVS	32

MV Liv syrup	Syrup	1	100	PAM	CVC (liver)	33

MV Liv tablet	Tablet	1	60	PAM	CVC (liver)	34

Pilarin	Tablet	1	90	PAM	GUS	35

Piles cure oil	Syrup	1	40	PAM	GUS	36

Pittghna churna	Powder	1	60	CAM	CVC (liver)	37

Sarivadhyasava	Syrup	1	90	CAM	DER	38

Shivastrim	Powder	1	5	PAM	DIG	39

Slim tea	Powder	1	50	PAM	NVS	40

Tonoliv	Syrup	1	45	PAM	CVC (liver)	41

Trinity	Tablet	1	50	PAM	CVC (liver)	42

Vata raktahar churna	Powder	1	70	CAM	CVC (blood)	43

Yakrit rasayan churna	Powder	1	70	CAM	CVC (liver)	44

Yuktadi churna	Powder	1	2	CAM	MSK	45

PAM = Proprietary Ayurvedic Medicine, CAM = Classical Ayurvedic Medicine.

CVC: Cardiovascular, DER: Dermatological, DIG: Digestive, GUS: Genitourinal, MSK: Musculoskeletal, NVS: Nervous, OTH: Others, and RES: Respiratory (following [29])

*Note: The liver is included for cardiovascular disease category because liver filters or detoxifies all the blood coming from the digestive tract before the blood circulates back into the heart. In human body, liver is the only organ having two blood supplies: hepatic artery bringing blood from heart and hepatic portal vein brining blood from the intestines.*

**Table 5:** Major use of ayurvedic medicines containing *Neopicrorhiza* as reported by industrial respondents and confirmed in market and industrial surveys.

**Ayurvedic medicines containing *Neopicrorhiza***	**Use of ayurvedic medicines containing *Neopicrorhiza* to treat illness associated with**	**Number of ayurvedic medicines containing *Neopicrorhiza***	**Percentage**
Heptogen capsule, Pittghna churna, Rohitkyadi churna, Yakrit rasayan churna, Hepadex, Hepagard DS, Hepatop, Heptogen syrup, Livergen, Livherb, Livorin, Livosave, Livotop, MV Liv syrup, Tonoliv, MV Liv tablet, Trinity	CVC (liver)	17	37.78

Glowderm, Madhumeha churna, Vata raktahar churna, Diabeno, Jameda Churna, Maha sudarshan churna	CVC (blood)	6	13.33

Jeevan shakti prash, Mana, Megaferol, Ayurved shakti churna, Arogyavardini vati, Slim tea	NVS	6	13.33

Jatyadi ghrita, Haridrakhanda, Neembadi churna, Sarivadhyasava	DER	4	8.89

Yuktadi churna, Dardnasak oil, Mahayogaraj guggul	MSK	3	6.67

Lachhadi oil, Pilarin, Kumaryasava, Piles cure oil	GUS	4	8.89

Kasarin, Asthamarin	RES	2	4.44

Gastro, Shivastrim	DIG	2	4.44

Kustadi danta manjan	OTH	1	2.22

Source: Field survey (2015/16)

CVC: Cardiovascular, DER: Dermatological, DIG: Digestive, GUS: Genitourinal, MSK: Musculoskeletal, NVS: Nervous, OTH: Others, and RES: Respiratory (following [29]).

**Table 6:** Changes of legal provisions in trade and conservation of *Neopicrorhiza* by Government of Nepal.

**Government notice (source)**	**Enforced date**	**Remarks on *Neopicrorhiza***
Nepal Gazette, 2001	2058.09.16 (12.28.2001)	Ban of *Neopicrorhiza* for collection, use, sale, distribution, transport and export

Nepal Gazette, 2003	2060.08.01 (17.11.2003)	Lifted the ban of *Neopicrorhiza* for collection, utilization, sale, distribution, transport and export.
		Conditions for export
		requires clear taxonomic identification of the species and confirmation by the Department of Plant Resources Department of Forest will permit the export after assessing the availability of the species. Otherwise, the export of this species is prohibited.

Nepal Gazette, 2005	2062.06.10 (11.25.2005)	Fixed the royalty rate NRs 10/kg for *Neopicrorhiza*. NRs 500 royalty for issuing CITES certificate for sale

Nepal Gazette, 2015	2072.07.17 (03.11.2015)	Revised royalty rate is NRs 15/kg. NRs 1000 royalty for issuing CITES certificate for export of CITES listed species, including *Neopicrorhiza*

**Table 7:** Traded quantity and revenue collection of *Neopicrorhiza* (2010/11–2015/2016).

**Fiscal year (BS)**	**Fiscal year (AD)**	**Quantity (Kg)**	**Revenue (NRs)**	**Revenue (USD)**	**Source (Provinces)**
2067/68	2010/11	47218	710010	6893.301	4, 7, Karnali

2068/69	2011/12	21704	330810	3211.748	4, Karnali

2069/70	2012/13	10304	124215	1205.971	4, 7, Karnali

2070/71	2013/14	34019	489225	4749.757	4, Karnali

2071/72	2014/15	50432.9	788145	7651.893	4, 7, Karnali

2072/73	2015/16	60957.5	918540	8917.864	3, 4, Karnali

	Total	224635.4	3360945	32630.53	

Source: Department of Forest, Government of Nepal

BS: Bikram Samvat (the official calendar of Nepal), AD: Anno Domini (the Julian and Gregorian calendar), NRs: Nepalese Rupees, USD: Unites States Dollar; flat exchange rate: USD 1 = NRs 103.

## Data Availability

The data used to support the findings of this study are available from the corresponding author upon written request. The questionnaire used in industrial survey is available in Smith-Hall et al. [28].
